# Serum vitamin B12 and folate status among patients with chemotherapy treatment for advanced colorectal cancer

**DOI:** 10.1080/03009730903027172

**Published:** 2009-09-07

**Authors:** Per Byström, Karin Björkegren, Anders Larsson, Linda Johansson, Åke Berglund

**Affiliations:** ^1^Department of Oncology and Pathology, Karolinska University HospitalStockholmSweden; ^2^Flogsta VårdcentralUppsalaSweden; ^3^Department of Medical Sciences, Akademiska SjukhusetUppsalaSweden; ^4^Department of Oncology, Radiology and Clinical Immunology, Akademiska SjukhusetUppsalaSweden

**Keywords:** Chemotherapy, cobalamin, colorectal cancer, folate, homocysteine

## Abstract

**Background:**

There are conflicting results on the role of cobalamin and folate for epidemiology and carcinogenesis in colorectal cancer patients and the need of supplementation for prevention of chemotherapy toxicity.

**Patients and methods:**

Serum cobalamin, folate, and homocysteine were analysed before and during the treatment of 93 patients with advanced colorectal cancer (ACRC) with first-line chemotherapy treatment. This cohort was compared with a healthy control group of 224 individuals.

**Results:**

Patients with ACRC had similar cobalamin, folate, and homocysteine values as the healthy control group. There were no correlations between serum cobalamin, folate, and homocysteine values and objective response. There were no correlations to anaemia or other severe toxicity for cobalamin and homocysteine. A total of 12 patients had folate deficiency, and 10 of those suffered from severe toxicity (grade 3 or more). All patients had markedly increased folate values after 2 months of treatment. Folate and homocysteine did not predict patient outcome; however, patients with subclinically low cobalamin values (<300 pmol/L) had significant better overall survival and time to progression than patients with normal or high cobalamin values.

**Conclusion:**

Patients with ACRC seem to have fairly adequate cobalamin and folate status before and during chemotherapy treatment. This study indicates that ACRC patients receiving chemotherapy do not need supplementation with vitamin B12 and folate. A minor portion of the patients had folate deficiency, and most of those patients had severe toxicity. Patients with subclinically low cobalamin values had surprisingly better survival.

## Introduction

Vitamin B12 deficiency may cause genetic instability, and folate is essential for regenerating methionine, the methyl donor for DNA methylation, and for producing the metabolites for DNA synthesis. Inadequate availability of folate may contribute to abnormalities in DNA synthesis or repair, either of which may influence colon carcinogenesis. There are several studies which indicate that people with deficiency of vitamin B12 and folate have an increased risk for developing colorectal cancer (1–4). There are also cohort studies indicating decreased risk for colorectal cancer among patients with high intake of folate, dietary or as supplementation (5). Other authors do not find these correlations, and there might be a theoretical risk of potentiating the progression of an already established early neoplastic clone (e.g. colorectal adenoma) (6).

Pemetrexed (Alimta®) is a novel multi-target antifolate that inhibits several enzymes involved in DNA synthesis such as thymidylate synthase (TS), dihydrofolate reductase (DHFR), and glycinamide ribonucleotide formyltransferase (GARFT). Early studies showed a severe toxicity profile, but further studies performed in the US yielded a lower grade of toxicity. The reason for that was assumed to be folate supplementation in flour (7). Therefore Alimta is given together with folate and vitamin B12 supplementation (8).

In a randomized, prospective, phase III trial (Nordic VI), irinotecan (Campto®—topoisomerase 1 blocker) in combination with either bolus 5-FU/leucovorin or bolus/infused 5-FU/leucovorin was compared in patients with metastatic colorectal cancer (9). These two schedules (FLIRI versus Lv5FU2-IRI) resulted in the same progression-free survival and overall survival, being the primary end-points, although the Lv5FU2-IRI schedule resulted in slightly more objective responses and slightly less toxicity. We have retrospectively analysed the patients from the two largest hospitals in the study for various clinical and other predictors of toxicity.

The aim of this study was to compare serum cobalamin, folate, and homocysteine values of ACRC patients before and during chemotherapy treatment, to detect any possible deficiency and to relate that to outcome and toxicity in a retrospective analysis.

## Material and methods

### Patients

From June 2001 to March 2004, 567 patients with non-resectable metastatic disease from histologically confirmed colorectal adenocarcinoma were included in a Nordic multi-centre study, the Nordic VI study. At two major hospitals, Uppsala and Stockholm, Sweden, 93 patients were included for serum sampling of cobalamin, folate, and homocysteine. This sub-study with B12, folate, and homocysteine status among ACRC patients was compared with a previously performed study on healthy volunteers in the same county with median age of 77 years (10).

No prior chemotherapy other than adjuvant 5-FU-based chemotherapy completed at least 6 months before the study entry was allowed. All patients should have measurable disease according to the response evaluation criteria in solid tumours (RECIST) (11), age between 19 and 76 years, WHO performance status of 0 to 2, and adequate laboratory values. The upper serum bilirubin level should be <1.25×the upper normal limit (UNL) (1.5 if liver metastases).

**Table I.** Patient characteristics.

Age, years	Median (range)	63 (42–76)
Colon		57
Rectum		36
Sex	Male	59
	Female	34
WHO	0	72
	1	18
	2	3
Primary tumour resected	Yes	72
	No	17
	Missing data	4
Adjuvant treatment	Yes	13
	No	80
Haemoglobin, g/L	Mean (range)	127 (94–158)
	Median	128
Objective response	Complete remission	5
	Partial remission	36
	Stable disease	35
	Progressive disease	12
	Not evaluable	5

The study was approved by the ethical committees at each site. All patients provided informed consent for the clinical study and for this sub-study.

### Treatment

The patients were treated in a multi-centre phase III study comparing two different fluorouracil (5-FU) schedules in combination with irinotecan. The patients were randomly assigned to receive irinotecan with the Nordic FLv schedule (12) or the Lv5FU2 schedule (13). The FLIRI regimen (14) consisted of irinotecan (Campto®) 180 mg/m^2^ (initially 210 mg/m^2^) as a 60-minute intravenous (i.v.) infusion on day 1, followed immediately by 5-FU 500 mg/m^2^ as a bolus (<5 min) injection, followed 30–40 minutes later by FA 60 mg/m^2^ i.v. bolus. The 5-FU/FA administrations were repeated on day 2. The Lv5FU2-IRI regimen (15) consisted of irinotecan 180 mg/m^2^ on day 1, followed by FA 200 mg/m^2^ as a 2-hour infusion, a 5-FU bolus of 400 mg/m^2^, followed by a 600 mg/m^2^ 22-hour infusion. The 5-FU/FA administrations were repeated on day 2. All treatments were repeated every 2 weeks until disease progression or unacceptable toxicity. A 20% reduction in dose of irinotecan and 5-FU was made in case of grade 3–4 haematological and non-haematological toxicities. A second 20% reduction was also allowed if toxicities remained. Treatments were delayed for up to 2 weeks if the haematological and gastrointestinal toxicities had not resumed for grade 1 at the time of next cycle.

Concomitant medication included subcutaneous atropine 0.25 mg, administered for cholinergic symptoms, oral loperamide 2 mg for diarrhoea, and, in case of fever or severe neutropenia, a prophylactic broad-spectrum oral antibiotic (ciprofloxacin) was to be administered. Patients with febrile neutropenia were hospitalized to receive i.v. antibiotics. The patients also received anti-emetic treatment with metoclopramide and 5-HT3 blockers.

### Response and toxicity evaluation

Tumour response was assessed according to RECIST criteria (11). Toxicity was evaluated according to the National Cancer Institute common toxicity criteria, version 2 (16). The information prospectively recorded in the case record forms was used.

### Serum sampling and measurements

Serum for cobalamin, folate, and homocysteine analyses was taken at base-line and prior to the fourth cycle, i.e. at 2 months of treatment. Immediately after sampling, aliquots were frozen at -20°C for later analyses. The results of the analyses were unknown for the treating physicians. Serum cobalamin (reagent: 6C09), folate (reagent: 7K60-32), and homocysteine (reagent: FHER100) were analysed on an Architect Ci8200 (Abbott Laboratories, Abbott Park, IL, USA) and reported using SI units. The total coefficients of variation were 7.0% at 480 pmol/L for cobalamin, 2.6% at 28 nmol/L for folate, and 2.7% at 29.3 µmol/L for homocysteine. The same measurement procedure was performed for the historical control of the healthy population.

### Statistical analyses

Comparisons of proportion between groups were performed by chi-square analyses. Correlations were studied with Spearman's rank correlation test. Survival curves were drawn according to Kaplan-Meier.

## Results

Patient homocysteine values did not correlate to patient outcomes, i.e. objective response (OR), time to progression (TTP), overall survival (OS), anaemia, or chemotherapy toxicity (data not shown).

**Table II.** The results of the analyses.

	n	Mean	Median	Range	95% CI
S-Cobalamin base-line, pmol/L	81	421	368	37–1476	134–853
S-Cobalamin after 2 months, pmol/L	67	409	354	13–1480	39–1123
S-Folate at base-line, nmol/L	93	10.0	7.6	1.0–45.3	3.0–30.4
S-Folate after 2 months, nmol/L	71	28.4	28.8	10.2–45.5	16.8–38.6
S-Homocysteine at base-line, µmol/L	93	19.3	17.8	6.6–43.5	9.6–36.2
S-Homocysteine after 2 months, µmol/L	73	16.6	13.5	0.6–230.0	8.2–21.4

CI = confidence intervals.

**Figure 1.**
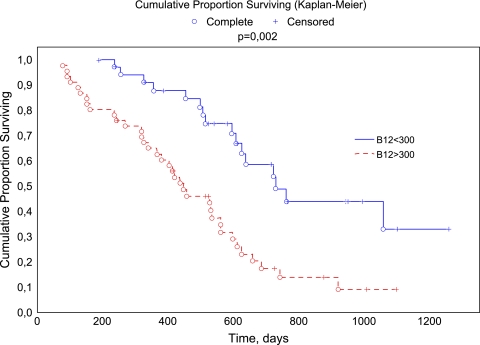
Overall survival curve according to base-line B12 status.

There were only two patients with cobalamin values lower than reference. Those 2 patients and 33 patients with subclinically low values (<300 pmol/L) did not have more toxicity than patients with higher values (approximately 50% of the patients suffered from at least one reported grade 3 or more toxicity). There was no correlation between cobalamin values and objective response; however, patients with values <300 pmol/L at base-line had better OS (*P =*0.0002) (Figure 1) and TTP (*P =*0.03) than patients with higher values. There were only modest changes of the patients’ cobalamin values after 2 months of treatment.

A total of 12 patients (13%) had folate deficiency before the start of the chemotherapy treatment, and, of those, 10 patients (83%) suffered from grade 3 toxicity or more. Those 10 patients had the same toxic profile as other patients with severe toxicity. All patients had markedly increased folate values after 2 months of chemotherapy treatment. There were no correlations with patients’ folate status and OR, TTP, or OS.

None of these markers correlated to haemoglobin value or performance status.

The patients’ cobalamin, folate, and homocysteine values were almost equal to a previously performed study with a healthy control group of 224 individuals from the same county (Table III), although the control group was slightly older (median 77 years).

**Table III.** Healthy controls (*n*=224).

	Mean	Median	95% CI
S-Cobalamin, pmol/L	376	302	342–410
S-Folate, nmol/L	11.5	8.8	10.2–12.8
S-Homocysteine, µmol/L	16.8	14.7	15.9–17.8

CI = confidence intervals.

## Discussion

In the current study, we examined serum levels of cobalamin, folate, and homocysteine in patients with ACRC, before and after 2 months of treatment.

There are, going through the literature, conflicting results on how to interpret the importance of the endogenous levels of B12, folate, and homocysteine in association with ACRC development, as discussed in the introduction, but also whether or not these levels should be taken into account when it comes to treatment.

5-Fluorouracil (5-FU) has been the mainstay of treatment of patients with ACRC and remains an integral component of modern combination regimens. Antifolate chemotherapy agents such as 5-FU reduce proliferation of neoplastic cells by inhibiting DNA synthesis. 5-FU interferes with the folate metabolism by inhibition of thymidylate synthetase. Interrupting the action of this enzyme blocks synthesis of the pyrimidine thymidine, which is a nucleotide required for DNA replication. Thymidylate synthase methylates deoxyuridine monophoshate (dUMP) into thymidine monophosphate (dTMP).

It has been postulated that measuring the levels of some of the key components (B12, folate, and homocysteine) in the folate metabolism would be of importance for tailored treatments for our patients. A number of clinical studies have illustrated that pretreatment levels of folate and homocysteine are predictive for both tumour response and toxicity (low folate levels are associated with increased toxicity) in patients treated with either thymidylate synthase (TS) inhibitors or folate antagonists (8,17,18). Previous murine studies have indicated that folate levels may predict for the cytotoxic efficacy of 5-FU (19). It has also been shown that an increased amount of exogenous folate leads to enhanced TS inhibition (20).

On the other hand, two recent clinical studies have indicated a relationship between dietary folate intake and fluoropyrimidine toxicity (21,22). In one study, 86 Canadian patients receiving adjuvant 5-FU and leucovorin were prospectively assessed for biomarkers of folate metabolism. Multivariate analyses identified base-line serum folate as an independent positive predictor for grade 3–4 toxicity. A similar result was found in a study of capecitabine monotherapy, in which patients with higher base-line levels of serum folate had a significantly increased incidence of toxic events (23).

These conflicting findings have been debated, and one explanation may be the different mechanisms of action of the antifolates. The antifolates compete with naturally occurring folate for active moieties on folate-dependent enzymes. Depletion in the level of intracellular folate will therefore lead to increased toxicity. Furthermore, the level of intracellular folate feeds back to inhibit the polyglutamation of antifolates, limiting toxicity. This, however, remains to be further elucidated.

In this study we have shown that base-line levels of B12, folate, and homocysteine for a patient population with advanced ACRC (*n*=93) were almost the same as for a previous performed cohort study of 224 healthy individuals from the same county. Our study also reveals that most individuals in an unselected population with advanced ACRC, in spite of their possible intestinal problems and earlier operations, still have normal levels of serum cobalamin, folate, and homocysteine. Cobalamin deficiency is rare in this population, and this is most likely explained by the fact that the endogenous stores of B12 last for several years, and the course of the disease normally is shorter. None of the two patients who had B12 levels below the reference experienced increased toxicity. On the contrary, 10 out of 12 patients who suffered from grade 3–4 toxicity had folate levels below the reference before start of treatment. This is, given the excess amount of folate administered as part of the treatment, somewhat doubtful to relate to the subclinically low levels of folate before the start of treatment in this population. A partial explanation might be the rate-limiting transport mechanism from extra- to intracellular space carried out by reduced folate carriers. Another explanation might be that the patients with the lowest levels of folate might be the ones with the most advanced disease and therefore the most undernourished.

Interestingly in this study, patients with subclinically low cobalamin values had superior overall survival and time to progression than did patients with higher values. It is not possible to offer a good explanation for this, but it might reflect a worse outcome among patients with more liver metastases. It is known that patients with liver injuries can have increased serum cobalamin values. Theoretically, patients with lower cobalamin values should have inferior outcome, i.e. with malnutrition etc. It could also be explained with a statistical mass effect.

Based on the results from this study, it is not clinically relevant to tailor treatment for individual ACRC patients based on B12, folate, or homocysteine levels. Most likely the individual patients gain more if we tailor treatment with respect to more established predictive markers for toxicity and treatment effect, like DPD, TS, and TP levels when treating with antifolates.

## References

[1] Duthie SJ, Narayanan S, Sharp L, Little J, Basten G, Powers H (2004). Folate, DNA stability and colo-rectal neoplasia. Proc Nutr Soc..

[2] Ryan BM, Weir DG (2001). Relevance of folate metabolism in the pathogenesis of colorectal cancer. J Lab Clin Med..

[3] Talley NJ, Chute CG, Larson DE, Epstein R, Lydick EG, Melton LJ 3rd (1989). Risk for colorectal adenocarcinoma in pernicious anemia. A population-based cohort study. Ann Intern Med..

[4] Prinz-Langenohl R, Fohr I, Pietrzik K (2001). Beneficial role for folate in the prevention of colorectal and breast cancer. Eur J Nutr..

[5] Giovannucci E, Stampfer MJ, Colditz GA, Hunter DJ, Fuchs C, Rosner BA (1998). Multivitamin use, folate, and colon cancer in women in the Nurses’ Health Study. Ann Intern Med..

[6] Kim YI (2004). Will mandatory folic acid fortification prevent or promote cancer?. Am J Clin Nutr..

[7] Midgley R, Kerr DJ (2009). Capecitabine: have we got the dose right?. Nat Clin Pract Oncol..

[8] Bunn P, Paoletti P, Niyikiza C, Rusthoven J, Nelson K, Hanauske AR (2001). Vitamin B12 and folate reduce toxicity of Alimta (pemetrexed disodium, Ly231514, MTA), a novel antifolate/antimetabolite. Proc Am Soc Clin Oncol.

[9] Glimelius B, Sorbye H, Balteskard L, Bystrom P, Pfeiffer P, Tveit KM (2008). A randomized phase III multicenter trial comparing irinotecan in combination with the Nordic bolus 5-FU and folinic acid schedule or the bolus/infused de Gramont schedule (Lv5FU2) in patients with metastatic colorectal cancer. Ann Oncol..

[10] Bjorkegren K, Svardsudd K (2001). Serum cobalamin, folate, methylmalonic acid and total homocysteine as vitamin B12 and folate tissue deficiency markers amongst elderly Swedes—a population-based study. J Intern Med..

[11] Therasse P, Arbuck SG, Eisenhauer EA, Wanders J, Kaplan RS, Rubinstein L (2000). New guidelines to evaluate the response to treatment in solid tumors. European Organization for Research and Treatment of Cancer, National Cancer Institute of the United States, National Cancer Institute of Canada. J Natl Cancer Inst..

[12] Glimelius B (1993). Biochemical modulation of 5-fluorouracil: A randomized comparison of sequential methotrexate, 5-fluorouracil and leucovorin versus sequential 5-fluorouracil and leucovorin in patients with advanced symptomatic colorectal cancer. Nordic Gastrointestinal Tumor Adjuvant Therapy Group. Ann Oncol..

[13] de Gramont A, Bosset JF, Milan C, Rougier P, Bouché O, Etienne PL (1997). Randomized trial comparing monthly low-dose leucovorin and fluorouracil bolus with bimonthly high-dose leucovorin and fluorouracil bolus plus continuous infusion for advanced colorectal cancer: a French intergroup study. J Clin Oncol..

[14] Glimelius B, Ristamaki R, Kjaer M, Pfeiffer P, Skovsgaard T, Tveit KM (2002). Irinotecan combined with bolus 5-fluorouracil and folinic acid Nordic schedule as first-line therapy in advanced colorectal cancer. Ann Oncol..

[15] Douillard JY, Cunningham D, Roth AD, Navarro M, James RD, Karasek P (2000). Irinotecan combined with fluorouracil compared with fluorouracil alone as first-line treatment for metastatic colorectal cancer: a multicentre randomised trial. Lancet..

[16] National Cancer Institute National Cancer Institute common toxicity criteria. http://ctep.cancer.gov/reporting/ctc.html.

[17] Calvert H (2002). Folate status and the safety profile of antifolates. Semin Oncol..

[18] Niyikiza C, Baker SD, Seitz DE, Walling JM, Nelson K, Rusthoven JJ (2002). Homocysteine and methylmalonic acid: markers to predict and avoid toxicity from pemetrexed therapy. Mol Cancer Ther..

[19] van der Wilt CL, Backus HH, Smid K, Comijn L, Veerman G, Wouters D (2001). Modulation of both endogenous folates and thymidine enhance the therapeutic efficacy of thymidylate synthase inhibitors. Cancer Res..

[20] Pinedo HM, Peters GF (1988). Fluorouracil: biochemistry and pharmacology. J Clin Oncol..

[21] Ho C, Ng K, O'Reilly S, Gill S (2005). Outcomes in elderly patients with advanced colorectal cancer treated with capecitabine: a population-based analysis. Clin Colorectal Cancer..

[22] Sharma R, Rivory L, Beale P, Ong S, Horvath L, Clarke SJ (2006). A phase II study of fixed-dose capecitabine and assessment of predictors of toxicity in patients with advanced/metastatic colorectal cancer. Br J Cancer..

[23] Lokich J (2004). Capecitabine: fixed daily dose and continuous (noncyclic) dosing schedule. Cancer Invest..

